# SHMT2 regulates serine metabolism to promote the progression and immunosuppression of papillary renal cell carcinoma

**DOI:** 10.3389/fonc.2022.914332

**Published:** 2022-08-30

**Authors:** Weiyu Kong, Zhongyuan Wang, Nuoran Chen, Yiwen Mei, Yang Li, Yulin Yue

**Affiliations:** ^1^ Department of Clinical Laboratory, Children’s Hospital of Nanjing Medical University, Nanjing, China; ^2^ First Clinical Medical College of Nanjing Medical University, Nanjing, China; ^3^ The State Key Lab of Reproductive, Department of Urology, The First Affiliated Hospital of Nanjing Medical University, Nanjing, China; ^4^ Department of Urology, Shanghai Pudong Hospital, Fudan University Pudong Medical Center, Shanghai, China

**Keywords:** cancer metabolism, serine, tumour microenvironment, SHMT2, immunotherapy

## Abstract

Recent research has demonstrated the diverse relationship between tumour metabolism and the tumour microenvironment (TME), for example, abnormal serine metabolism. This study investigated the role of serine metabolism in papillary renal cell carcinoma (pRCC) focusing on the prognostic value and regulatory mechanisms. Gene expression profiles and clinical data of patients with pRCC were obtained from The Cancer Genome Atlas (TCGA) database and Gene Expression Omnibus (GEO) database. Kaplan–Meier curves were used for survival analysis and consensus clustering for tumour serine metabolic signatures extraction. Functional analysis, including the Kyoto Encyclopedia of Genes and Genomes (KEGG) and gene set enrichment analysis (GSEA), was applied to explore the biological characteristics. The gene set variation analysis (GSVA), single-sample GSEA (ssGSEA), and Estimation of Stromal and Immune cells in Malignant Tumour tissues using Expression data (ESTIMATE) methods were utilised to estimate the immune infiltration in the various subtypes. Five serine metabolic genes (SMGs) were used to classify patients with pRCC, with four clusters identified with diverse prognoses and immune features based on these survival-related SMGs. Further analysis of the best and worst clusters (B and D clusters) revealed variations in survival, clinical progression, oncogenic pathways, and TME, which included immune infiltration scores, immunosuppressive cell infiltration, and expression of immune checkpoints. In addition, SMGs, especially SHMT2, exacerbated the carcinogenesis and immunosuppressive cells in pRCC, thus promoting tumour proliferation. In conclusion, higher SHMT2 gene expression and higher serine metabolism in tumour cells are associated with poorer clinical outcomes in pRCC. SHMT2 is a potential novel target gene for targeted therapy and immunotherapy in pRCC.

## Introduction

Abrupt changes in metabolism frequently occur during tumourigenesis and tumour progression (1, 2). This major shift depends on the metabolic reorganisation of cancer cells. Owing to higher demands for bioenergy and biosynthesis, cancer cells normally alter their metabolic pathways (3), initiated by cancer-driven gene mutations and the effectiveness of environmental nutrients (4). Under these circumstances, abnormal metabolic accumulation occurs, which influences tumour microenvironment (TME) (5, 6), thus promoting the tumour. Therefore, exploring the regulatory mechanism of tumour metabolic abnormalities is essential for targeted cancer treatment.

Among these metabolic pathways, the amino acid–related pathways have received wide attention, such as serine that has important implications in tumourigenesis (7). Serine supplies a one-carbon unit when converted to glycine (8, 9), which then participates in the methionine cycle, either generating the primary methyl donor S-adenosylmethionine (SAM) or involved in ATP synthesis (10). Serine metabolism is also vital in redox homeostasis. When serine is converted to glycine, it indirectly promotes the synthesis of GSH, a major redox regulator (11). Cancer cells require NAD(P)H-reductive equivalents (12, 13). Serine could also provide NAD(P)+ in the folate cycle, thus acting as a tumour facilitator (14). Many studies have revealed an increased concentration of serine in breast cancer and melanoma due to excessively activated serine anabolism (15), but serine metabolism has not been investigated in papillary renal cell carcinoma (pRCC). Therefore, this study systematically evaluated the serine metabolic atlas in pRCC and classified patients into four subtypes, which represented different prognostic and microenvironment characteristics. In addition, we filtered the most vital serine metabolism regulators in pRCC, which were closely related to the immune microenvironment and immune checkpoints. The metabolic subtypes and SHMT2 expression levels are promising novel targets for immunotherapy.

## Methods

### Data acquisition

The comprehensive transcriptome expression matrix of patients with pRCC together with the clinical data was extracted from The Cancer Genome Atlas (TCGA-KIRP; https://www.cancer.gov/tcga) (**Table 1**). Five genes associated with serine metabolic pathways identified in a previous study (14) were defined as serine metabolic genes (SMGs). Three datasets (GSE26574 (16), GSE2748 (17), and NIHMS1737783 (18)) were acquired from the Gene Expression Omnibus (GEO; http://www.ncbi.nlm.nih.gov/geo/) database and National Center for Biotechnology Information to validate the consistency between the key gene SHMT2 and pRCC clinical outcomes, and the immune microenvironment and immune checkpoints. The dataset NIHMS1737783 was submitted by Motzer et al. and included 886 patients’ clinical data and 726 tissue samples (18). The Human Protein Atlas (HPA) database (https://www.proteinatlas.org) was utilised to analyse the protein expression in pRCC and normal tissues.

**Table 1:** Clinical characteristics of included patients in the study.

Variables	Total (n = 289)	Cluster A (n = 100)	Cluster B (n = 68)	Cluster C (n = 90)	Cluster D (n = 31)
Age (year)					
<65	173 (59.86%)	54 (54.00%)	38 (55.88%)	57 (63.33%)	24 (77.42%)
≥65	113 (39.10%)	45 (45.00%)	30 (44.12%)	31 (34.44%)	7 (22.58%)
Unknown	3 (1.03%)	1 (1.00%)	0 (0.00%)	2 (2.22%)	0 (0.00%)
Gender					
Female	77 (26.64%)	39 (39.00%)	10 (14.71%)	13 (14.44%)	15 (48.39%)
Male	212 (73.36%)	61 (61.00%)	58 (85.29%)	77 (85.56%)	16 (51.61%)
Stage					
I	172 (59.52%)	50 (50.00%)	45 (66.18%)	65 (72.22%)	12 (38.71%)
II	22 (7.61%)	8 (8.00%)	3 (4.41%)	6 (6.67%)	5 (16.13%)
III	51 (17.65%)	20 (20.00%)	11 (16.18%)	11 (12.22%)	9 (29.03%)
IV	15 (5.19%)	10 (10.00%)	1 (1.47%)	1 (1.11%)	3 (9.68%)
Unknown	29 (10.03%)	12 (12.00%)	8 (11.76%)	7 (7.78%)	2 (6.45%)
T stage					
T1	193 (66.78%)	58 (58.00%)	51 (75.00%)	72 (80.00%)	12 (38.71%)
T2	33 (11.42%)	13 (13.00%)	5 (7.35%)	8 (8.89%)	7 (22.58%)
T3	59 (20.42%)	28 (28.00%)	11 (16.18%)	9 (10.00%)	11 (35.48%)
T4	2 (0.69%)	0 (0.00%)	1 (1.47%)	1 (1.11%)	1 (3.23%)
TX	2 (0.69%)	1 (1.00%)	0 (0.00%)	0 (0.00%)	0 (0.00%)
N stage					
N0	49 (16.96%)	16 (16.00%)	10 (14.71%)	17 (18.89%)	6 (19.35%)
N1	24 (8.30%)	9 (9.00%)	4 (5.88%)	5 (5.56%)	6 (19.35%)
N2	4 (1.38%)	4 (4.00%)	0 (0.00%)	0 (0.00%)	0 (0.00%)
NX	211 (73.01%)	70 (70.00%)	54 (79.41%)	68 (75.56%)	19 (61.29%)
Unknown	1 (0.35%)	1 (1.00%)	0 (0.00%)	0 (0.00%)	0 (0.00%)
M stage					
M0	95 (32.87%)	42 (42.00%)	15 (22.06%)	30 (33.33%)	8 (25.81%)
M1	9 (3.11%)	6 (6.00%)	0 (0.00%)	0 (0.00%)	3 (9.68%)
MX	171 (59.17%)	47 (47.00%)	48 (70.59%)	57 (63.33%)	19 (61.29%)
Unknown	14 (4.84%)	5 (5.00%)	5 (7.35%)	3 (3.33%)	1 (3.23%)

### Differential gene analysis

The Wilcoxon test and R package *Limma* were used to detect variations in SMGs expression between tumour and normal samples. The significance criterion was set as a false discovery rate (FDR) <0.05 and an absolute value of log2 fold change >1.5.

### Survival analysis

To obtain the best cutoff point to classify samples into high and low expression groups, the “surv_cutpoint” command was utilised according to the mRNA expression. Then, a log-rank test was applied to examine the outcome results by the Kaplan–Meier method. R packages *KMsurv*, *survival*, and *survminer* were used to conduct prognostic analysis. A P-value <0.05 was considered statistically significant.

### Cluster analysis

The subtypes of pRCC samples were obtained by the R package *ConsensusClusterPlus (* 19). The datasets were clustered by the Euclidean squared distance metric and the K-means algorithm with k from 2 to 9. The results were displayed in the form of heatmaps of the consistency matrix using the R package *pheatmap*. The most optimal subtypes were screened following these criteria: high consistency of clustering, a moderate sample size for each cluster, a significant difference in survival rate and clinical characteristics, and no significant increase in area under the cumulative distribution function (CDF) curve.

### Immune microenvironment assessment

Estimation of the Stromal and Immune cells in Malignant Tumour tissues using Expression data (ESTIMATE) analysis was performed to quantify the TME of each sample with R package *estimate*, including the ESTIMATE score, immune score, stromal score, and tumour purity. Single-sample gene set enrichment analysis (ssGSEA) was used to calculate the relative abundance of immune cells with the R package *GSVA* based on the gene sets from the study of Charoentong et al. (20) The Student’s t-test was used to evaluate differences in the above parameters between clusters, and a P-value <0.05 was defined as statistically significant.

### Biological pathway enrichment analysis

Gene set variation analysis (GSVA) was performed to evaluate pathway enrichment for different clusters with the R package *GSVA* and “c2.cp.kegg.v7.4.symbols” from the Molecular Signatures Database. On the basis of the Kyoto Encyclopedia of Genes and Genomes (KEGG) database (http://www.genome.jp/kegg), enrichment of KEGG pathways was performed by R package *clusterProfiler*. Pathways characterised by a nominal P-value <0.05 and a FDR <0.05 were identified as significant pathways.

### Correlation analysis

On the basis of the SHMT2 median expression, the pRCC samples were classified into SHMT2 high expression and low expression groups. The correlation analysis of the key gene expression with clinical characteristics was performed in the TCGA-KIRP samples and was validated in the GSE26574 and GSE2748 cohorts. To identify the relationship between SHMT2 expression and the TME, ESTIMATE and the ssGSEA algorithm were used to analyse the proportion of tumour-infiltrating immune subsets. The Pearson correlation coefficient (R) and P-value were calculated, in which |R| > 0.3 and p < 0.05 were considered statistically significant.

### Verification of expression level

The expression of SHMT2 at the translation level was validated by the HPA online database (https://www.proteinatlas.org/). The freely available HPA database provides the protein expression profiles, as well as immunohistochemistry (IHC) images for a wide variety of cancer tissues. The IHC analysis in the HPA database is also presented for many protein-coding genes in patients with respective cancer, and the antibody information used for each IHC analysis can also be obtained in the HPA database. The IHC score is mainly classified into strong, moderate, weak, and negative based on the staining intensity and fraction of stained cells (21, 22).

### Response prediction of anti-tumour drugs

To evaluate SHMT2 for pRCC treatment, the IC50 of commonly administered anti-tumour drugs in the TCGA project of the pRCC dataset was calculated using the algorithm developed by Geeleher et al. (23) and the related R package pRRophetic (24). This algorithm can create statistical models from the gene expression and drug sensitivity data from cell lines in the Cancer Genome Project, which allows users to predict the clinical therapeutic response using only the baseline tumour gene expression data. The guidelines of the American Joint Committee on Cancer recommend 30 common anti-tumour drugs, such as Axitinib, Bortezomib, and Elesclomol, for cancer treatment. The difference in the IC50s of these common anti-tumour drugs between the high– and low–SHMT2 expression groups was compared, and the results are shown as box plots. The Wilcoxon rank sum test was conducted, and p < 0.05 was considered statistically significant.

### Statistical analysis

All analyses were performed using R 4.1.3. All statistical tests were two-sided, and a P-value <0.05 was considered statistically significant unless otherwise noted. Continuous variables that conformed to normal distribution were compared using an independent t-test for comparison between groups, whereas continuous variables with skewed distribution were compared with the Mann–Whitney U-test. The relationship between hub genes and overall survival (OS) was analysed through the Kaplan–Meier curve that was evaluated by a log-rank test.

## Results

### The expression pattern and prognostic effect of serine metabolism genes in pRCC

**Figure 1:**
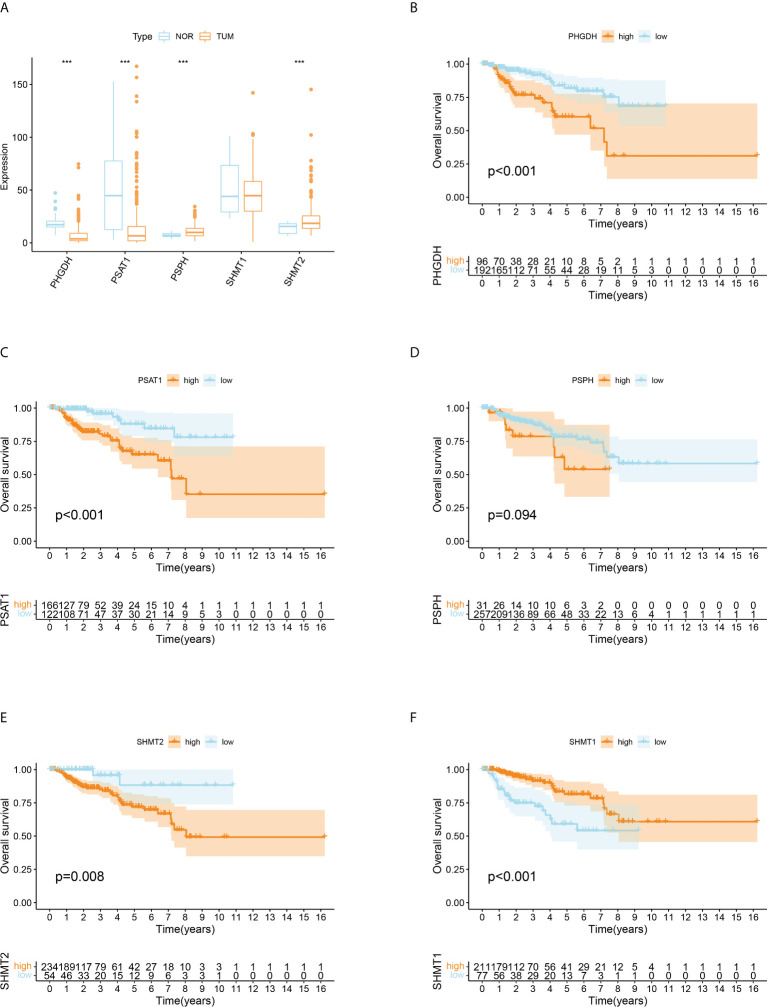
Differentially expressed serine metabolic genes (SMGs) in papillary renal cell carcinoma (pRCC) and normal tissues and their relationship with prognosis. **(A)** The expression profiles of SMGs in pRCC and normal tissues. Yellow, tumour; blue, normal. The upper and lower ends of the boxes represent the interquartile ranges, and the lines in the boxes represent the median values. Adjusted p < 0.05 and |log2 fold changes (FC)| > 1.5 were used as the criteria for screening differentially expressed SMGs; *p < 0.05, **p < 0.01, and ***p < 0.001. **(B–F)** Survival analyses for each differentially expressed SMG based on 289 patients with pRCC from TCGA cohorts. Kaplan–Meier curves with log-rank p < 0.05 showed a significant survival difference between the high expression and low expression groups. The shaded area represents the 95% confidence interval.

The levels of intracellular serine are regulated by various genes participating in its synthesis, transformation, and transport. A total of five serine metabolism genes were used in this study, of which four had significantly different expression between tumour and normal tissues (**Figure 1A**). The expression of PSPH and SHMT2 was significantly higher in tumour tissues, whereas PHGDH and PSAT1 were reduced. An estimation of the relationship between SMGs and the prognosis of patients with pRCC revealed that a higher expression of PHGDH, PSAT1, PSPH, and SHMT2 was significantly associated with worse clinical outcomes (**Figures 1B–E**). On the contrary, a higher expression of SHMT1 was closely related to better OS (**Figure 1F**). These findings suggested that the serine metabolism genes had aberrant expression in pRCC and may play distinctive roles in tumourigenesis.

### Serine metabolism subtypes with distinct prognosis and biological features in pRCC

**Figure 2:**
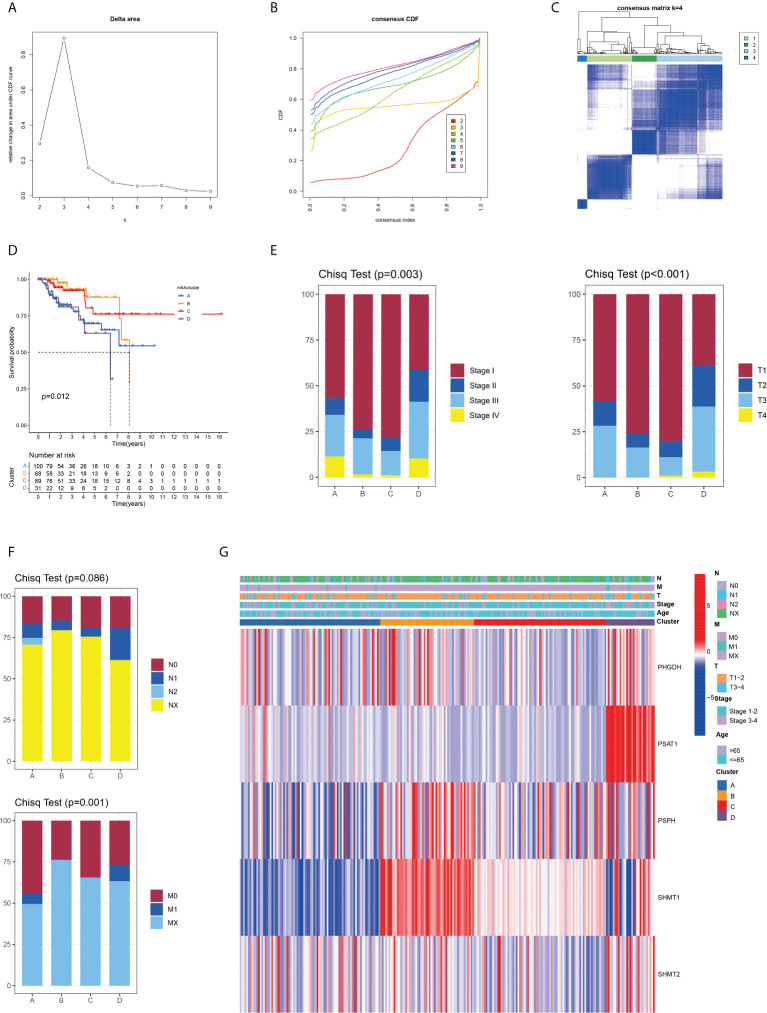
Identification and clinical correlation of various serine metabolic molecular patterns of pRCC. **(A)** Delta region graph showing the relative change in the area under the CDF curve for K = 2–9. **(B)** Consensus clustering cumulative distribution function (CDF) for K = 2–9. **(C)** Consensus clustering matrix for K = 4. **(D)** Kaplan–Meier overall survival (OS) curve for 289 patients with pRCC in the cluster A/B/C/D subgroups. Patients in cluster B had the best OS (p = 0.012). **(E, F)** Difference in the proportion of cases with different pathological stages (Chisq Test, p = 0.003), T stage (Chisq Test, p < 0.001), M stage (Chisq Test, p = 0.001), and N stage (Chisq Test, p = 0.086) from TCGA among A, B, C, and D clusters. **(G)** On the basis of the results of the cluster analysis, the heatmap showed the correlation with clinicopathological characteristics. Red represents a high expression of SMGs, and blue represents a low expression.

**Figure 3:**
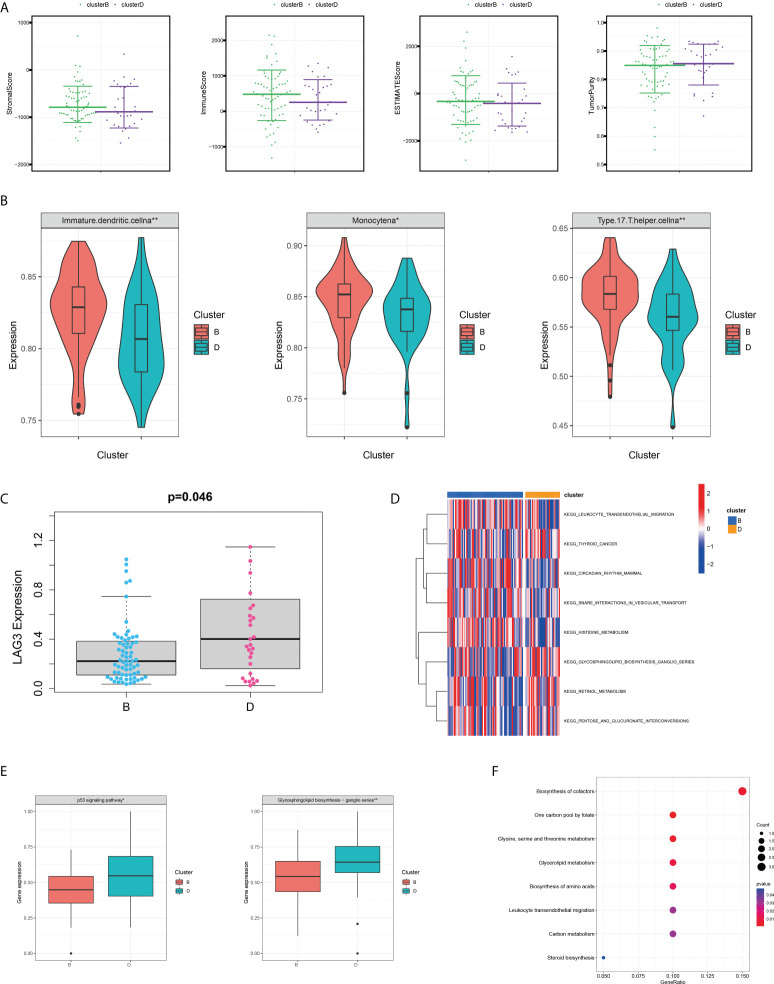
Variations of TME immune-related characteristics and metabolic molecular patterns between cluster B and D. **(A)** Samples in cluster B demonstrated higher stromal, immune, and ESTIMATE scores and lower tumour purity than those in cluster D. **(B)** The abundance of the main immune-infiltrating cells in TME in the two serine metabolic patterns. Cluster D was classified as the immunosuppressive phenotype, characterised by immune suppression. **(C)** Differences in the expression of LAG3, one common immune checkpoint, between clusters B and D (P = 0.046). **(D)** GSVA enrichment analysis revealed the activation level of biological pathways in distinct serine metabolic modification patterns. The heatmap was utilised for visualisation, in which red represents activated pathways and blue represents inhibited pathways. **(E)** Differences in tumour-related pathways including the p53 signalling pathway and glycosphingolipid biosynthesis pathway between the two serine metabolic patterns; *p < 0.05, **p < 0.01, and ***p < 0.001. **(F)** KEGG pathway enrichment of differentially expressed genes between clusters B and D. The improved items were analysed using gene counts, gene ratio, and adjusted p-values.

Given that all five SMGs might have synergistic effects in patients with pRCC, a cluster analysis was conducted on the basis of the expression of these SMGs. As shown in **Figure 2A**, k = 4 was considered optimal, as confirmed by the CDF plot and delta area plot (**Figures 2B, C**). To test the correctness of the clustering results, the prognostic correlation was evaluated by Kaplan–Meier curves (**Figure 2D**). The differences in clinical stage composition between the different groups are shown in **Figures 2E, F**. The proportion of patients with advanced clinical stages was higher in both group A and group D, which indicated a higher susceptibility to the development of end-stage tumours. The general clinicopathological characteristics were illustrated by the heatmap (**Figure 2G**). To further investigate the biological mechanism of prognostic divergences between different subgroups, two extreme subtypes (cluster B: n = 68 and cluster D: n = 31) were selected for further study. The ESTIMATE analysis showed that patients in cluster B had a higher StromalScore, ImmuneScore, and ESTIMATEScore. Likewise, there was higher tumour purity in patients in cluster D that represented a lower ESTIMATEScore (**Figure 3A**). Furthermore, immune cell infiltration analysis demonstrated that effector immune cells including dendritic cells, monocytes, and type 17 T helper cells were more enriched in cluster B than that in cluster D, leading to better immune scores in cluster B (**Figure 3B**). Moreover, the expression of immune checkpoints LAG3 was significantly higher in cluster D (**Figure 3C**). These results indicated that cluster D represented an immunosuppressive group, which might be the reason for the poor prognosis. In **Figure 3D**, the GSVA results showed that gene sets in cluster B were significantly enriched in snare interactions in the vesicular transport pathway and histidine metabolism pathway. Notably, GSEA analysis revealed that the p53 signalling pathway and glycosphingolipid biosynthesis were enriched in cluster D, both of which contributed to tumour proliferation (**Figure 3E**). As further validation, KEGG enrichment analysis suggested that the dysregulated genes in cluster D mainly participated in cellular processes and environmental information processing pathways, including biosynthesis of cofactors; one-carbon pool by folate; and glycine, serine, and threonine metabolism (**Figure 3F**).

### Clinical and prognostic features of SHMT2 in the TCGA database and GEO validation datasets

**Figure 4:**
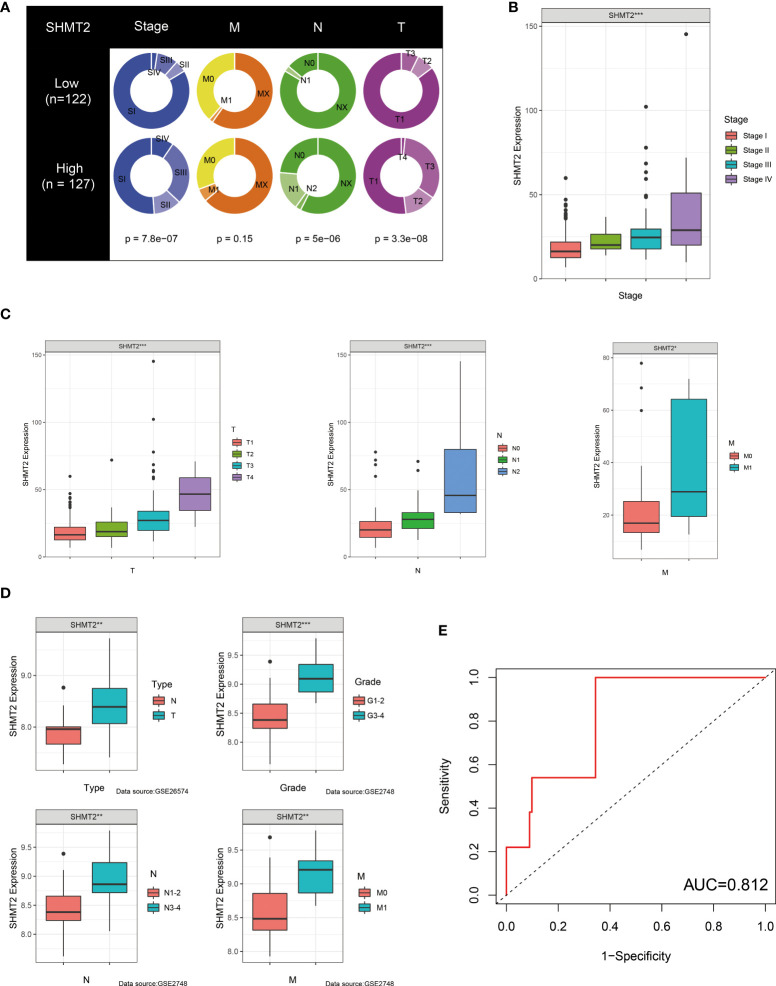
SHMT2 expression is upregulated in advanced pRCC. **(A)** According to data from TCGA, patients with pRCC with high SHMT2 expression tended to be in a more severe disease state, including pathological stage, T stage, and N stage. **(B, C)** There was a significant positive correlation between SHMT2 expression and the pathological stage, T stage, N stage, and M stage. **(D)** Validation of the relationships between SHMT2 expression and clinical parameters in the GSE26574 and GSE2748 datasets. **(E)** Time-dependent ROC analysis of the TCGA-pRCC set. The AUC value of 0.812 suggested that the forecasting ability of SHMT2 expression was sensitive; *p < 0.05, **p < 0.01, and ***p < 0.001.

Because of its significant overexpression in tumour tissues and poor prognosis in survival analysis, it was hypothesised that SHMT2 might play a role in the tumourigenesis and progression of pRCC (**Figures 1A, E**). To demonstrate the accuracy of a single-gene SHMT2 prognostic prediction, clinical samples were divided into high and low groups with median SHMT2 expression as the criteria. Samples with unknown messages were deleted. The pie charts exhibited different proportions of clinical characteristic distribution and suggested that patients in the high–SHMT2 expression group tended to be in a more advanced disease stage, such as pathological stage (p < 0.001), T stage (p < 0.001), and N stage (p < 0.001) (**Figure 4A**). An evaluation of the correlation between SHMT2 expression and clinical predictors revealed that, with the cumulative amount of SHMT2 in tumour cells, the tumour would further deteriorate in different aspects, including pathological stage (p < 0.001), T stage (p < 0.001), N stage (p < 0.001), and M stage (p < 0.05) (**Figures 4B, C**). In addition, data from GSE26574 showed higher SHMT2 expression in tumour cells than that in normal cells, whereas data from GSE2748 revealed a consistent relationship between SHMT2 expression and clinical predictors (**Figure 4D**). To confirm whether the expression of SHMT2 was a reliable prognostic indicator, the Receiver Operating Characteristic (ROC) was plotted and the AUC value was 0.812, which meant that SHMT2 expression had good sensitivity and specificity in predicting tumourigenesis (**Figure 4E**).

### The potential role of SHMT2 in the tumour immune microenvironment

**Figure 5:**
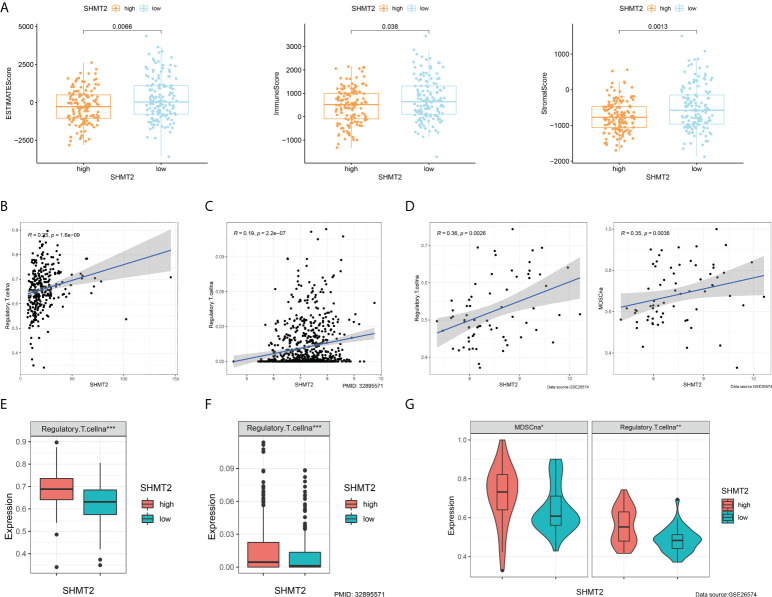
SHMT2 was associated with the immunosuppressive microenvironment of pRCC. **(A)** Correlation analyses of SHMT2 expression with tumour-infiltrating immune cells (TICs) from the ESTIMATE algorithm. High SHMT2 expression was associated with low ImmuneScores (p = 0.038), StromalScores (p = 0.0066), and ESTIMATEScores (p = 0.0013). **(B–D)** Correlation analyses of SHMT2 expression with the proportion of primary immunosuppressive cells in **(B)** TCGA samples, **(C)** NIHMS1737783 samples, and **(D)** GSE26574 samples. **(E, F)** High SHMT2 expression corresponded with significantly higher Treg cell infiltration in **(E)** TCGA samples, **(F)** NIHMS1737783 samples, and **(G)** GSE26574 samples. In addition, MDSCs were more easily enriched in the high–SHMT2 expression subgroup; *p < 0.05, **p < 0.01, and ***p < 0.001.

**Figure 6:**
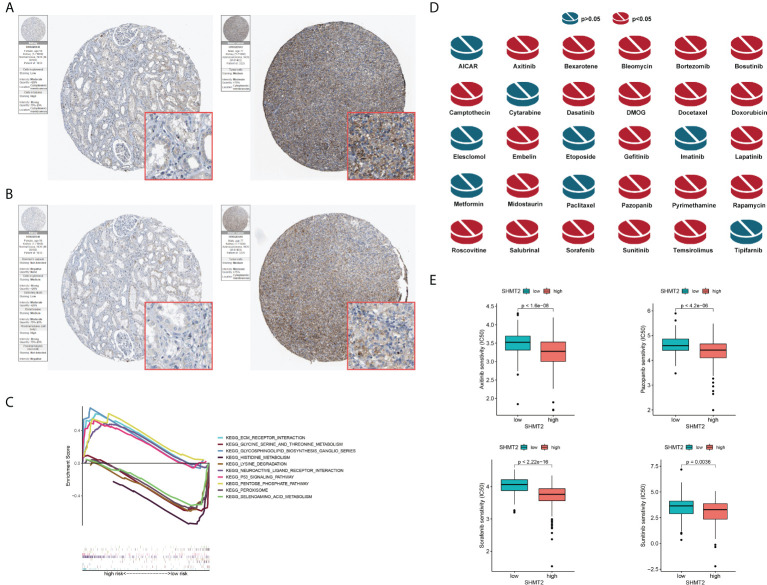
Validation of the role of SHMT2 at the translational level and in anti-tumour drugs. **(A, B)** Immunohistochemical images from the HPA database show SHMT2 protein expression in normal kidney (pictures on the right) and pRCC (pictures on the left) tissues by different antibodies. **(C)** GSEA analysis of KEGG in the high– and low–SHMT2 expression subgroups. Pathways such as the ECM receptor interaction pathway and p53 signalling pathway were upregulated in high–SHMT2 expression subgroups, which are recognised as the oncogenic pathways. **(D)** Estimated drug sensitivity in patients with high and low SHMT2 expression. The IC50s of 22 anti-tumour drugs were significantly different in the different SHMT2 expression groups. **(E)** The difference of anti-tumour drugs in IC50, including sunitinib (p = 0.0036), sorafenib (p < 2.22e-16), axitinib (p < 1.6e-08), and pazopanib (p < 4.2e-06) between the high– and low–SHMT2 expression subgroups. IC50, the half inhibitory concentration, and a P-value < 0.05 was considered statistically significant.

To further examine the relationship between SHMT2 and TME, correlation analyses of SHMT2 expression with tumour-infiltrating immune cells (TICs) from the ESTIMATE algorithm and ssGSEA signatures were performed. The ESTIMATE results showed that patients with high SHMT2 expression had a higher StromalScore (p = 0.038), ImmuneScore (p = 0.0013), and ESTIMATEScore(p = 0.0066; **Figure 5A**), representing the larger amount of the immune or stromal components in the TME. **Figures 5B–D** show the results of ssGSEA from different data sources, suggesting that SHMT2 was significantly positively correlated with the infiltration of regulatory T cells (Tregs) (TCGA: R = 0.35, p < 0.001; NIHMS1737783: R = 0.19, p < 0.001; GSE26574: R = 0.36, p = 0.0026) and myeloid-derived suppressor cells (MDSCs) (GSE26574: R = 0.35, p = 0.0036). Consistent with the results above, there were significantly more infiltrated immunosuppressive cells in high SHMT2 expression samples in the TCGA, NIHMS1737783, and GSE26574 cohorts (**Figures 5E–G**). The HPA database was used to confirm the protein expression in pRCC and normal tissues. In IHC images, SHMT2 was expressed more in tumour tissues and mainly located in the cytoplasm of pRCC cells. The comparison of staining with two different antibodies “HPA020543” and “HPA020549” is displayed in **Figures 6A, B**. In the KEGG analysis, the p53 signalling pathway; glycine, serine, and threonine metabolism pathway; and extracellular matrix (ECM) receptor interaction pathway were proved enriched in the high–SHMT2 expression group (**Figure 6C**).

### Comparison of the sensitivity to anti-tumour drugs between patients with different SHMT2 expression levels

Clinical decisions should be based on the different molecular subtypes of pRCC; therefore, sensitivity to the 30 common anti-tumour drugs was compared between the high– and low–SHMT2 expression groups to determine potential treatment modalities for pRCC. The results demonstrated that the IC50s of the 22 anti-tumour drugs were significantly different in the two SHMT2 expression groups (**Figure 6D**). Furthermore, the IC50s of axitinib (p < 0.001), pazopanib (p < 0.001), sorafenib (p < 0.001), and sunitinib (p = 0.0036), the four recommended anti-tumour drugs for the renal cell carcinoma treatment, were lower in patients with higher SHMT2 expression, which suggests that increased SHMT2 expression level was accompanied by increased sensitivity to these four drugs (**Figure 6E**). In this context, these drugs have the potential to be applied in the treatment of high SHMT2 expression pRCC in the future.

## Discussion

During tumourigenesis and proliferation, tumour cells tend to reshape the surrounding environment, which is defined as reprogramming of TME (25). The rapid growth of tumour cells results in a relatively hypoxic cellular environment (26), leading to tumour cells utilising anaerobic glycolysis as their main metabolic mode. Excessive hydrogen ions in the tumour cells drain into the extracellular matrix to form an acidic, oxygen-deprived TME. The TME not only creates good conditions for tumour cell growth but also inhibits immune cell infiltration and activation. Furthermore, it has been shown that amino acid metabolism is second only to glucose metabolism in the development of tumours. An adequate amino acid supply provides tumour cells with sufficient amounts of anabolic substrates, thereby promoting tumour growth. In this sense, studies of amino acid metabolism may reveal the internal molecular events of malignant tumours and provide new ideas for tumour diagnosis and treatment.

As a non-essential amino acid, serine has been proved to be enriched in tumour cells (27, 28). The one-carbon unit produced during the conversion of serine to glycine is the preferred carbon source for tumour cells, eventually becoming SAM through the folate cycle and involved in nucleotide synthesis (29). Simultaneously, the synthesis of serine is essential for maintaining cell redox homeostasis (30). Herein, serine metabolism was shown to be closely related to the occurrence, development, and prognosis of pRCC. In Dr. Linehan’s study, different types of patients with pRCC display variant metabolic alterations, including glycolysis, Krebs cycle, electron transport chain, and ribose metabolism. Meanwhile, the variability in metabolism also leads to different OS (31). Similarly, our study focused on serine metabolism that was closely related to these metabolic pathways described above. According to the transcriptome profiling, four pRCC subtypes were defined with distinct clinical and biological characteristics. In essence, through the *de novo* synthesis of serine, tumour cells could express proteins and synthesise lipids more easily. The conversion of serine to glycine provides substrates for nucleotide synthesis. In lymphoma, the upregulation of the serine synthesis pathway is a metabolic marker of B-cell activation and the germinal centre response. Overexpression of enzymes involved in serine synthesis is a feature of germinal centre B-cell–derived lymphoma (31). In addition, PSAT1 activation by PERK could promote macrophage immunosuppressive activity through serine biosynthesis and regulate the efficacy of immunotherapy in melanoma (32). Furthermore, monoubiquitination-mediated PHGDH activity enhancement promoted serine synthesis and one-carbon unit metabolism and increased the content of intracellular SAM, thus accelerating CRC liver metastasis (33). The findings above suggest that serine metabolism participates in the progression of various malignant tumours.

In the present study, the bioinformatic analysis revealed a clear increase in SHMT2 expression in human pRCC samples, which served as the most potent regulator of serine metabolism. In previous research, the SHMT2 gene was defined as an oncogene, which exerted its function in mitochondria (34), mediating the conversion of serine to glycine and tetrahydrofolate (THF) to N5, N10-methylated THF (MTHF). MTHF is the main transportation form of the one-carbon unit and contributes to the metabolism of proteins and nucleotides metabolism, which corresponds with tumour cell growth. In the folate cycle, MTHF is converted to formylated THF required for the formylation of the initial methionine on mitochondrial transfer RNAs for mitochondrial protein translation. Li et al. demonstrated that SIRT5 could increase the activity of SHMT2 by desuccinylation modification, thus enhancing the serine metabolism and promoting the rapid proliferation of tumour cells (35). This study described possible therapeutic strategies to inhibit tumour cell proliferation by restraining serine metabolism. Similarly, another study showed that the deacetylase SIRT3 could remove the acetylation modification of SHMT2 K95, thus stabilising the intracellular expression of SHMT2 and maintaining the high activity of SHMT2 (36). The high activity of SHMT2 could help cells resist the pressure of reactive oxygen species in the mitochondria and ensure the supply of biological macromolecules in cells, thereby aggravating the malignant proliferation of cancer cells. In addition, another study described that SHMT2 and BCL2 cooperated to promote the occurrence of lymphoma through the silencing of epigenetic inhibitors, as the enhancement of SHMT2 metabolic enzyme activity was sufficient to convert normal B cells into B-cell lymphoma (37). In general, the increase of SHMT expression or activity drives serine metabolism and supports cancer cell proliferation by supplying nucleotides, which may become a novel molecular therapeutic target.

Furthermore, we found that SHMT2 may serve as an immune regulator to promote the remodelling of immunosuppressive TME. A growing number of studies have shown that tumour metabolism not only plays a key role in maintaining tumour progression and metastasis but also remodelled the immune microenvironment by releasing metabolites (38). Specifically, metabolites produced by tumour cells were released into the TME, which could interfere with the metabolic programme of immune cells, ultimately leading to the emergence of an immunosuppressive microenvironment. Similarly, Luo et al. showed that the expression of SHMT2 was associated with tumour-infiltrating lymphocytes, including activated type 1 T helper cells and natural killer cells (39). Notably, the present study demonstrated that SHMT2 could promote the infiltration of Treg cells and mediate immune escape. Tregs are a subset of lymphocytes that maintain peripheral tolerance and can induce antigen tolerance to tumour cells through immunosuppression, which indirectly accelerates tumour cell proliferation. Specifically, activated Treg cells destroy tumour immunity by enriching a series of costimulatory molecules (such as ICOS, CD27, 41BB, OX40, and GITR) and immune checkpoints (PD-1, CTLA-4, LAG3, and TIGIT) to promote tumour immune evasion (40–42). Currently, there was no report on SHMT2 regulating Treg infiltration. Kurniawan et al. explored the impact of serine metabolism on Treg function (43), showing that *de novo* serine synthesis in Tregs interfered with Foxp3 expression. Restriction of serine availability by GSH preserved FoxP3 expression and Treg function. Recently, immunotherapy is being increasingly applied for cancer treatment, but the reshaping of TME makes tumour tissues resistant to immunotherapy. In Burkitt lymphoma, SHMT2 expression correlated significantly with the effect of immunotherapy (44). Our findings suggested that SHMT2 may be associated with the immunotherapy response in patients with renal cancer; therefore, SHMT2 may be a potential novel target for combination immunotherapy.

This study has some limitations. The results need to be further validated by *in vitro* experiments, such as quantitative real-time polymerase chain reaction and Western blotting. Meanwhile, studies on human tissue samples are indispensable. Moreover, the biological mechanism of SHMT2 reconstructing the TME needs to be explored *in vivo* and *in vitro*.

## Conclusion

In conclusion, we investigated the potential role of serine metabolism in pRCC. Further, we explored the impact of metabolism reprogramming on the remodelling of the immune microenvironment in pRCC based on the data analysis of transcriptomics. Moreover, we demonstrated that the probable immunosuppressive role of SHMT2 in TME may be a promising therapeutic target in pRCC. Ultimately, serine metabolism as a target of tumour therapy needs to be further explored in the animal models and preclinical studies.

## Data availability statement

The original contributions presented in the study are included in the article/supplementary material. Further inquiries can be directed to the corresponding authors.

## Authors contributions

YY and YL designed this work. WK and NC wrote the manuscript. ZW performed the bioinformatics analysis. YM performed the data review. All authors contributed to the article and approved the submitted version.

## Conflict of interest

The authors declare that the research was conducted in the absence of any commercial or financial relationships that could be construed as a potential conflict of interest.

## Publisher’s note

All claims expressed in this article are solely those of the authors and do not necessarily represent those of their affiliated organizations, or those of the publisher, the editors and the reviewers. Any product that may be evaluated in this article, or claim that may be made by its manufacturer, is not guaranteed or endorsed by the publisher.
